# Enhancing Safety in Hyperbaric Environments through Analysis of Autonomic Nervous System Responses: A Comparison of Dry and Humid Conditions

**DOI:** 10.3390/s23115289

**Published:** 2023-06-02

**Authors:** Carlos Sánchez, Alberto Hernando, Juan Bolea, David Izquierdo, Germán Rodríguez, Agustín Olea, María Teresa Lozano, María Dolores Peláez-Coca

**Affiliations:** 1 BSICoS Group, I3A Institute, IIS Aragón, University of Zaragoza, 50018 Zaragoza, Spain; alberto.hernandosanz@ioon.es (A.H.); maytelo@unizar.es (M.T.L.); mdpelaez@unizar.es (M.D.P.-C.); 2 Departamento de Física, Centro Universitario de la Defensa de Zaragoza, Academia General Militar, 50090 Zaragoza, Spain; jbolea@unizar.es; 3 GTF Group, I3A Institute, University of Zaragoza, 50018 Zaragoza, Spain; d.izquierdo@unizar.es; 4 Departamento de Ingeniería y Técnicas Aplicadas, Centro Universitario de la Defensa de San Javier, Academia General del Aire, 30729 Murcia, Spain; german.rodriguez@cud.upct.es; 5 Centro de Buceo de la Armada de Cartagena, 30205 Murcia, Spain; aoleag@fn.mde.es

**Keywords:** hyperbaric environments, autonomic nervous system, heart rate variability, safety ranges, bagging method

## Abstract

Diving can have significant cardiovascular effects on the human body and increase the risk of developing cardiac health issues. This study aimed to investigate the autonomic nervous system (ANS) responses of healthy individuals during simulated dives in hyperbaric chambers and explore the effects of the humid environment on these responses. Electrocardiographic- and heart-rate-variability (HRV)-derived indices were analyzed, and their statistical ranges were compared at different depths during simulated immersions under dry and humid conditions. The results showed that humidity significantly affected the ANS responses of the subjects, leading to reduced parasympathetic activity and increased sympathetic dominance. The power of the high-frequency band of the HRV after removing the influence of respiration, $\bar{P_{HF⊥}}$, and the number of pairs of successive normal-to-normal intervals that differ by more than 50 ms divided by the total number of normal-to-normal intervals, $\bar{pNN50}$, indices were found to be the most informative in distinguishing the ANS responses of subjects between the two datasets. Additionally, the statistical ranges of the HRV indices were calculated, and the classification of subjects as “normal” or “abnormal” was determined based on these ranges. The results showed that the ranges were effective at identifying abnormal ANS responses, indicating the potential use of these ranges as a reference for monitoring the activity of divers and avoiding future immersions if many indices are out of the normal ranges. The bagging method was also used to include some variability in the datasets’ ranges, and the classification results showed that the ranges computed without proper bagging represent reality and its associated variability. Overall, this study provides valuable insights into the ANS responses of healthy individuals during simulated dives in hyperbaric chambers and the effects of humidity on these responses.

## 1. Introduction

Diving is an activity that can have significant cardiovascular effects on the human body, including changes in heart rate (HR), blood pressure and cardiac output, and alterations in pulmonary circulation [1]. These changes can place significant stress on the cardiovascular system and increase the risk of worsening underlying cardiomyopathies during diving. Therefore, it is important for individuals with preexisting cardiac conditions to undergo a thorough medical evaluation before engaging in diving activities.

Elevated environmental pressure causes changes in cardiac function, including an increase in systolic volume and a decrease in HR to maintain an adequate cardiac output and minimize the impact on the body. This adaptation is possible thanks to the autonomic nervous system (ANS) response [2]. Assessing the ANS response to significant pressure changes is challenging and may vary significantly among individuals. Studies aimed at monitoring and controlling this response in extreme conditions, as well as identifying potential health risks for divers, are of great interest. Such studies are particularly important in the training of military personnel. Typically, the response of the ANS is characterized by both time and frequency analysis of the heart rate variability signal (HRV), particularly those related to temporal changes in HR and those related to the power of the different spectral bands and their relationships. Previous studies have shown variations in these indices when the barometric pressure changes [3,4].

It is important to mention that there are serious health issues that can be derived from diving activities, particularly if the decompression protocol is not properly followed, that cannot be directly studied from the HRV signal. The clearest example is an arterial gas embolism, which can occur when air bubbles, usually caused by pulmonary barotrauma during a rapid ascent, enter the bloodstream and travel to the heart and other organs, leading to tissue damage and potentially life-threatening complications [5]. In addition, diving can also increase the risk of long-term cardiovascular problems such as arterial disease, hypertension, and atherosclerosis. According to a study published in the Journal of the American College of Cardiology, diving-induced stress on the cardiovascular system can lead to endothelial dysfunction, oxidative stress, and chronic inflammation, which can contribute to the development of cardiovascular diseases [6].

While increased pressure is a critical factor in the adaptive response of the ANS during diving, other variables can also significantly affect this response. The diving environment itself can influence the sympathovagal balance and the adaptation to humid conditions, known as the diving reflex. In this reflex, the HR slows down to reduce oxygen consumption, mediated by the parasympathetic nervous system. Cold water temperature leads to vasoconstriction, which is related to increased sympathetic activity. Additionally, the buoyancy and relaxation effects of water immersion promote parasympathetic activity.

Several other factors influence the sympathovagal balance during diving. Physical exertion and increased cardiovascular workload can trigger sympathetic activation. The respiratory pattern plays a role, with breathing through a regulator potentially inducing sympathetic activation, while controlled breathing techniques may promote vagal tone. Moreover, gas density is influenced by pressure and temperature, which can impact the ease and efficiency of breathing. Psychological factors, such as stress and anxiety, can also promote sympathetic activity [7]. Moreover, individuals with previous expertise in diving tend to experience relaxation and subsequent parasympathetic dominance.

The present study aims to investigate the autonomic response during diving in both dry and humid conditions, taking into account that factors related to the environment may also have an impact, and to identify HRV-derived indices that show significant discrepancies between the two conditions. For this purpose, electrocardiographic signals (ECG) were recorded from two groups of subjects. The first group performed a simulated immersion in a dry hyperbaric chamber, while the second group underwent the same immersion protocol in a humid hyperbaric chamber.

## 2. Materials and Methods

### 2.1. Databases

This study included two separate databases: one conducted in a dry hyperbaric chamber and the other in a humid hyperbaric chamber. The first database consists of 28 volunteers (25 males and 3 females), with a mean age of 28.5 ± 6.2 years. The second database includes 5 male volunteers with a mean age of 33.5 ± 2.4 years. All volunteers provided written informed consent, which was validated by the Ethics Committee ‘Comité de ética de la investigación con medicamentos de la inspección general de sanidad de la Defensa’.

ECG signals were recorded from all participants in the dry hyperbaric chamber at the Hospital General de la Defensa en Zaragoza (Spain) and in the humid hyperbaric chamber at the Centro de Buceo de la Armada in Cartagena (Spain). The Nautilus device, developed by the University of Kaunas, Lithuania [8], was used for this purpose. This device allowed us to record the ECG signal with three non-orthogonal leads at a sampling frequency of 2000 Hz, providing high-quality ECG data for analysis.

The hyperbaric immersion protocol lasted approximately two hours, during which time the pressure was gradually increased from 1 atm (reference pressure at sea level) to a maximum of 5 atm, with time intervals following the decompression table recommendations (see www.naui.org/resources/, accessed on 15 April 2023). During the immersion in both hyperbaric chambers, the participants underwent five stops, each lasting five minutes, at 1, 3, and 5 atm during both the descent (D) and ascent (A) phases. Specifically, the following stages were analyzed in this study: from “1D” (baseline state) to “3D”, “5” (maximum depth), “3A”, and back to “1A”. There were, however, some relevant differences in the positions of the subjects in each chamber. In the dry chamber, the subjects were seated quietly without performing abrupt movements during the whole immersion protocol. In the humid chamber, the subjects were lying down flat, submerged under water, breathing air from a compressed air bottle, wearing a wetsuit, and holding onto a bar with one hand to maintain the position during the whole immersion protocol.

### 2.2. Heart Rate Variability

To detect the position of heartbeats in the ECG signal, an algorithm based on the wavelet transform was used [9,10]. The algorithm corrected ectopic beats, missed beats, and false detections. Next, the instantaneous heart rate (HR) signal was computed using the integral pulse frequency modulation model at a sampling rate of 4 Hz [11]. In order to obtain the HRV signal, low-frequency modulation of HR (i.e., mean heart rate (HRM)) was subtracted by low-pass filtering at 0.03 Hz. Then, the HRV signal was calculated as the difference between the two terms: HRV=HR−HRM.

Out of all of the possible indices that could be used for the analysis of the ANS response, eight ECG-derived indices were selected from both the delineated ECG and the HRV signal to reflect the activity of the sympathetic and parasympathetic branches. Four indices were selected from the time domain and another four from the frequency domain.

### 2.3. Time Indices

**Figure 1:**
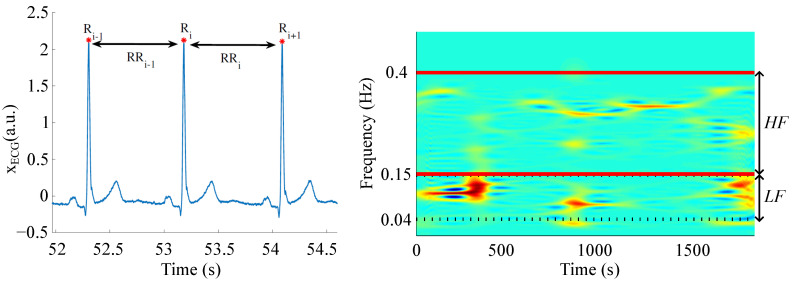
Example of an ECG signal with R peaks and the RR time series of two consecutive beats (**left panel**). Time-frequency map of the HRV signal (warm colours related to high power; cool colours related to low power) of a subject during the stage at 5 atm of the immersion with the HF and LF bands delimited with horizontal solid and dotted lines, respectively (**right panel**).

The four time-domain indices were computed from the R-wave interval series, and their averaged values in the last four minutes of each immersion stage were obtained. These indices were the following:

- The median of normal-to-normal intervals ($\bar{NN}\left(s\right)$), which correspond to the RR series (left panel in Figure 1) after removing ectopic beats:
  (1) $$\bar{NN}\left(s\right)=MED\left(NN\right)=Q2\left(NN\right).$$
- The interquartile range of NN intervals ($\bar{IQRNN}\left(s\right)$) as a measure of statistical dispersion:
  (2) $$\bar{IQRNN}\left(s\right)=IQR\left(NN\right)=Q3\left(NN\right)-Q1\left(NN\right).$$
- The root mean square of the successive differences between adjacent NN intervals ($\bar{RMSSD}\left(s\right)$):
  (3) $$\bar{RMSSD}\left(s\right)=\frac{\left||NN|\right|}{\sqrt{M}},$$
  where *M* represents the number of NN intervals.
- The number of pairs of successive NN intervals that differ by more than 50 ms divided by the total number of NN intervals ($\bar{pNN50}\left(\%\right)$), expressed as a percentage:
  (4) $$\bar{pNN50}\left(\%\right)=\frac{\sum_{i=1}^{M-1}p_{i}}{\sqrt{M-1}}*100,$$
  where $p_{i}=1$ if $|NN_{i+1}-NN_{i}|\;>50\;\mathrm{ms}$, $p_{i}=0$ if $|NN_{i+1}-NN_{i}|\;\leq 50\;\mathrm{ms}$, and *M* is the number of NN intervals.

### 2.4. Frequency Indices

One of the most common problems found in the analysis of ANS activity using classical indices in the frequency domain is the fact that the respiration frequency may lie within the low-frequency band of the HRV spectrum, which may mask the effects produced by other factors and, therefore, would lead to misleading conclusions [12]. The method used to avoid this issue in this study involves breaking down the HRV signal into two distinct components. The first component is associated with respiration and includes all variations that are linearly related to breathing. The second component, known as the residual component, includes all of the dynamics that are modulated by mechanisms other than respiration, including the sympathetic nervous system and other vagal modulators that may not be linked to respiration. Essentially, the residual component provides insight into the dynamics of the ANS that are not specifically related to respiration [13,14].

To apply this method, both respiratory and HRV signals are needed. Therefore, an ECG-derived respiration method based on the slopes and angles of the ECG signal was used to obtain the respiration signal [15,16]. In order to extract all of the dynamics of HR that are linearly related to respiration, the HRV is projected onto a subspace V defined by all variations in the respiratory signal. This subspace is constructed using the respiratory signal and its delayed versions, as shown in the literature [17]. Then, the HRV signal can be projected onto the respiratory subspace V obtaining a new signal denoted as $HRV_{R}$. The last step is to remove this projected signal from the original HRV to obtain an orthogonal component of it (known as a residual component) containing all of the effects unrelated to respiration as follows: $HRV_{⊥}=HRV-HRV_{R}$.

The powers of both the respiratory component ($\bar{P_{R}}\left(a.u.\right)$) and the residual component ($\bar{P_{⊥}}\left(a.u.\right)$), i.e., those corresponding to the HRV spectrum after removing respiration, were computed in this study:

(5) $$\bar{P_{R}}\left(a.u.\right)=\frac{HRV_{R}\cdot HRV_{R}}{HRV\cdot HRV},$$

(6) $$\bar{P_{⊥}}\left(a.u.\right)=\frac{HRV_{⊥}\cdot HRV_{⊥}}{HRV\cdot HRV}.$$

The other two analyzed frequency indices represented the power of the residual component in the LF band (0.04–0.15 Hz) reflecting the activity of the sympathetic system ($\bar{P_{LF⊥}}\left(a.u.\right)$) and the power of the residual component in the HF band (0.15–0.4 Hz) reflecting the activity of the parasympathetic system ($\bar{P_{HF⊥}}\left(a.u.\right)$) (right panel in Figure 1):

(7) $$\bar{P_{LF⊥}}\left(a.u.\right)=\int_{0.04}^{0.15}HRV_{⊥}\left(f\right)\;df,$$

(8) $$\bar{P_{HF⊥}}\left(a.u.\right)=\int_{0.15}^{0.4}HRV_{⊥}\left(f\right)\;df,$$

where $HRV_{⊥}\left(f\right)$ is the power spectrum of the residual component of the HRV signal.

**Figure 2:**
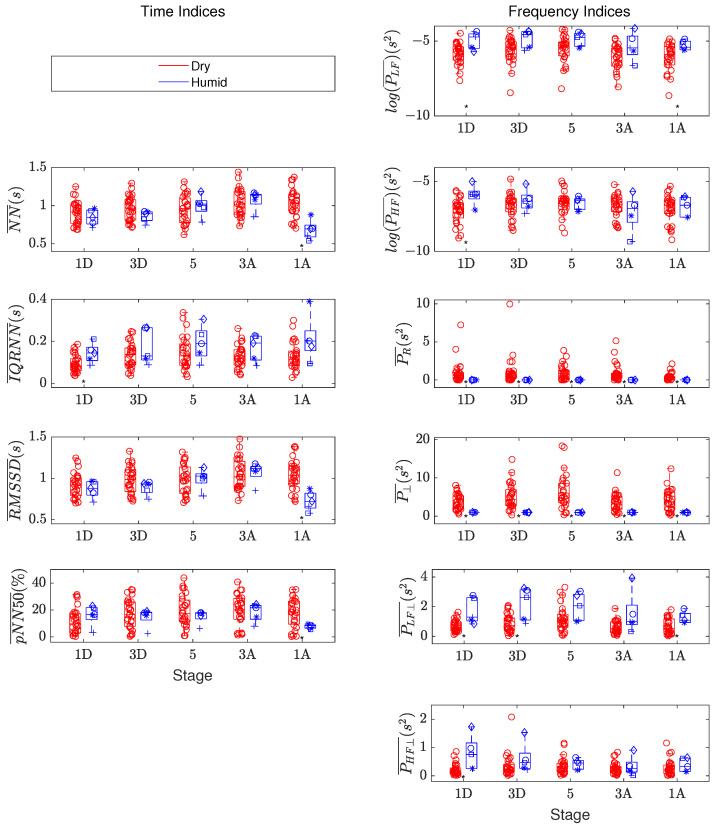
Boxplots of the computed values of the time indices (**left panels**) and frequency indices (**right panels**), including the logarithms of $\bar{P_{LF}}$ and $\bar{P_{HF}}$ directly extracted from the HRV spectrum, in the five stages of the immersion for each dataset: red for the dry hyperbaric chamber; blue for the humid hyperbaric chamber. The central line in each boxplot indicates the median value; the top and bottom edges are the first and third quartiles, respectively; and the vertical lines represent the minimum and maximum values not considered to be outliers (i.e., 1.5 IQR away from the bottom/top of the box). Individual values are plotted with symbols on top of the boxplots. *: *p*-value < 0.05 with Wilcoxon signed-rank test.

Figure 2 shows the values of the time indices (left panels) and frequency indices (right panels). Additionally, this figure includes the logarithm of $\bar{P_{LF}}$ and $\bar{P_{HF}}$ directly extracted from the HRV spectrum without using the orthogonal subspace projection, for both datasets (dry in red, humid in blue).

### 2.5. Statistical Analysis and Subject Classification

As observed in Figure 2, there are large variabilities between different subjects in the values of the indices extracted. Therefore, in order to focus on relative changes during the immersion, these indices were referenced to their values at the baseline stage “1D” using the following expression:

(9) $$R\left(\bar{Y_{S}}\right)=\frac{\bar{Y_{S}}-\bar{Y_{1D}}}{\bar{Y_{S}}+\bar{Y_{1D}}},$$

where $\bar{Y_{S}}$ is the value of the index computed at stage “S”, and $\bar{Y_{1D}}$ is the value of the index computed at the baseline stage “1D”. Note that only one value per index representative of the ANS response at each stage for each subject was computed. Since all of the computed indices after referencing them to the baseline have positive values, the interpretation of the indices using this equation is very intuitive: a value close to 1 means that the index value at that stage is much larger than the index measured at “1D”, a value close to −1 means the index value at that stage is much smaller than the index measured at “1D”, and a value around 0 means the index value at that stage has not changed much with respect to the index measured at “1D”.

In order to statistically compare the distribution of the indices in dry and humid hyperbaric chambers for each stage of immersion, Wilcoxon signed-rank test analyses were performed.

Due to the reduced number of subjects in the humid dataset, they were used as a “test group” to be classified according to the values obtained with the “train group” (i.e., the subjects of the dry dataset). To perform this classification, we first calculated the interquartile (IQR) and 5–95 percentile (%) ranges for each stage in the dry dataset, referred to as “safety-range models”. The classification of divers of the humid dataset was then based on the number of indices over a total of 32 (8 indices per stage for 4 stages) outside of these ranges, indicating abnormal responses during the immersion: more than 8 indices out of the 5–95 percentile ranges and/or more than 20 indices out of the IQR was considered as “abnormal”. This also enabled us to identify the indices with the greatest impact on the classification and the stages where more significant differences in the ANS response occurred.

## 3. Results

**Figure 3:**
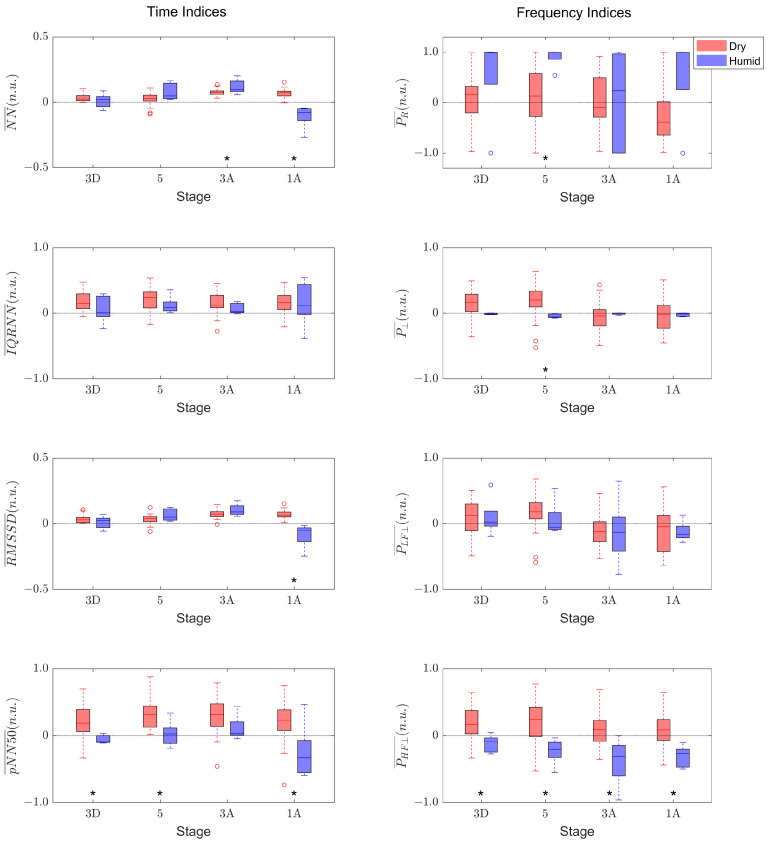
Boxplots of the normalized values of the time indices (**left panels**) and frequency indices (**right panels**) in the four stages of the immersion for each dataset: red for the dry hyperbaric chamber; blue for the humid hyperbaric chamber. The central line in each boxplot indicates the median value; the top and bottom edges are the first and third quartiles, respectively; and the vertical lines represent the minimum and maximum values not considered to be outliers (i.e., 1.5 IQR away from the bottom/top of the box). Outliers are shown with circles. *: *p*-value < 0.05 with Wilcoxon signed-rank test.

Figure 3 illustrates that the computed indices showed similar trends in the referenced indices between the two groups of subjects. To avoid unnecessary repetitions, the referencing symbol R is not used in the rest of the text of the manuscript when specific indices are mentioned. The time indices in the dry dataset exhibited positive values in general for all stages, with slight increases over time, whereas the trend was also increasing for the indices with the humid dataset, except for a strong decrease in the stage “1A” for $\bar{NN}$, $\bar{RMSSD}$, and $\bar{pNN50}$. Only the $\bar{pNN50}$ exhibited significant differences between the two datasets in three out of the four stages analyzed. Regarding the frequency indices, the general trends over time were not so clear, with the four indices showing median values close to 0 in the dry dataset, and similarly with $\bar{P_{⊥}}$ and $\bar{P_{LF⊥}}$ in the humid dataset. $\bar{P_{HF⊥}}$ displayed the most significant differences, since, in the humid dataset, it took negative values and showed a decreasing trend over time. It is worth mentioning that $\bar{P_{R}}$ had a large variability in both datasets, whereas that of $\bar{P_{⊥}}$ was very reduced, particularly in the humid dataset.

Interestingly, the stages that showed significant differences in the highest number of indices were the stage with the highest pressure, stage “5” (for $\bar{pNN50}$, $\bar{P_{R⊥}}$, $\bar{P_{⊥}}$ and $\bar{P_{HF⊥}}$), and the last stage of the immersion, stage “1A” (for $\bar{NN}$, $\bar{RMSSD}$, $\bar{pNN50}$, $\bar{P_{⊥}}$), which suggests that the differences between datasets were more prominent at high pressure and after spending a long time in hyperbaric conditions (see Figure 3).

### 3.1. Safety Ranges

**Figure 4:**
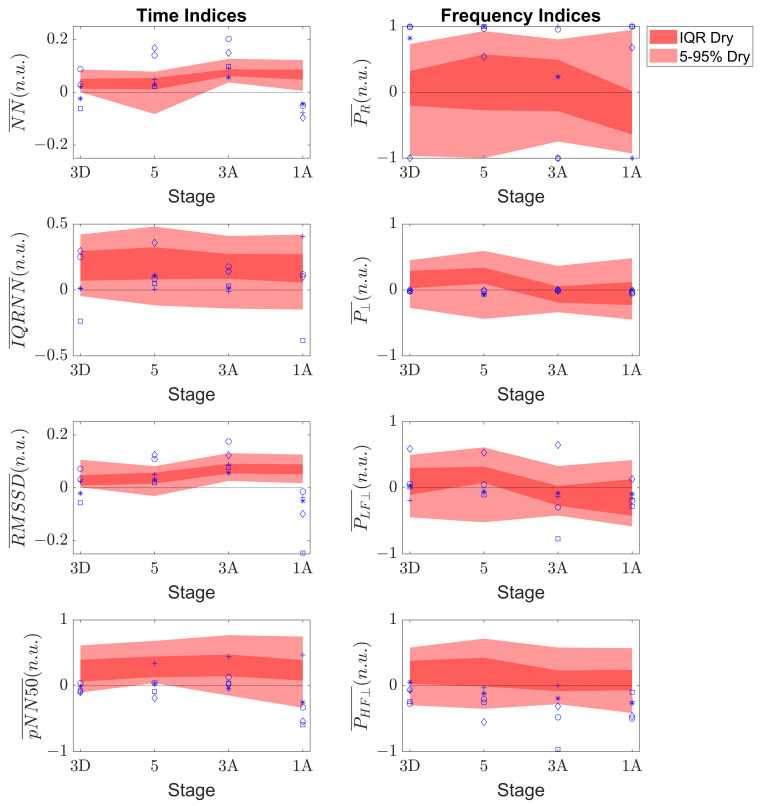
IQR (dark red) and 5–95 percentile ranges (light red) of the baseline-referenced values of the time indices (**left panels**) and frequency indices (**right panels**) in the four stages of the immersion for dry dataset. The five subjects of the humid dataset are shown with different symbols in blue.

Figure 4 shows the evolution of both the IQR and 5–95 percentile ranges in dry hyperbaric conditions (in red) together with the individual values of the five subjects of the humid dataset (symbols in blue) during the simulated immersions. It is worth mentioning that the index variations between consecutive stages were considered linear for the representation of the patches. As can be derived from the analysis of the statistical ranges, $\bar{NN}$ and $\bar{RMSSD}$ showed, in general, less prominent variations than the rest of the indices with respect to the baseline in all of the stages of immersion (i.e., the values were closer to 0). Interestingly, the ranges tended to be enlarged in the last stage of the immersion for all of the time indices, when the pressure was the same as in the baseline conditions before the immersion started, probably indicating that ANS recovery may be highly variable between subjects with different previous diving expertise and/or physical condition, whereas the widths of the ranges of the frequency indices were more stable in the four stages of immersion.

In the analysis of the humid indices with the dry chamber ranges, there was an overlap between the two groups for all of the stages and indices, except for $\bar{NN}$ and $\bar{RMSSD}$ in the “1A” stage. This was due to the narrow ranges of these two indices, indicating small relative variations between the subjects in both datasets. The differences in the two other time indices were subtle: the $\bar{IQRNN}$ trends were similar in both datasets, while $\bar{pNN50}$ showed larger positive ranges in the dry chamber subjects than in the humid chamber subjects in the early stages of immersion. This resulted in an increasing overlap between the two groups as the immersion progressed.

Regarding the frequency indices, the large variability of the $\bar{P_{R}}$ was reflected in both the wide ranges observed in Figure 4 and the indices of the humid subjects. The ranges of $\bar{P_{⊥}}$ overlapped; however, in this case, the overlap was due to the thin width of the ranges computed with the humid dataset. Regarding the ranges of $\bar{P_{LF⊥}}$ and $\bar{P_{HF⊥}}$, the main differences were observed in the second, with indices showing more negative values in the humid dataset than the dry dataset ranges, suggesting a reduction in the index during the immersion with respect to the baseline.

### 3.2. Subject Classification

As explained in the Methods section, the determination of whether a subject’s ANS response was classified as “abnormal” relied on the identification of indices that fell outside the previously calculated ranges. This classification process was conducted using all of the indices of individual subjects in both datasets (28 dry + 5 humid) with the ranges derived from the 28 subjects of the dry dataset. The results of the classification are shown in Table 1.

As expected, since the ranges were based on the dry dataset values, out of the 28 subjects of the dry chamber, none of them exhibited significantly abnormal behavior (i.e., with more than 8 indices outside of the 5–95 percentile dry ranges), and only 3 subjects showed more than 20 indices outside of the IQR (one female and two males). In the classification of the subjects in the humid chamber dataset, of the five subjects, two showed more than 8 indices outside of the 5–95 percentile range, one of them with more than 20 indices outside of the IQR.

### 3.3. Stage and Index Classification

**Figure 5:**
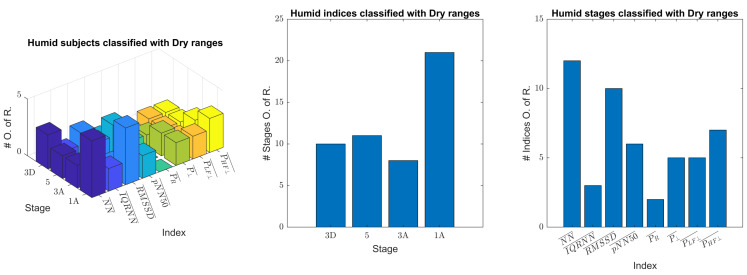
Total number of subjects per stage and index (**left panel**), indices per stage (**middle panel**), and stages per index (**right panel**) classified as out of range with the humid dataset and dry ranges.

In order to identify the stages and indices in which differences in the ANS behavior between both databases were more prominent, an additional classification of the humid subjects with the dry ranges was performed. Figure 5 shows the indices of the humid subjects, separated by stages, outside of the 5–95 percentile range of the dry database. On the one hand, the stage with the largest number of indices classified as out of range was “1A” (over 50% of a total number of indices of 40 (8 indices × 1 stage × 5 subjects)). On the other hand, the indices that led to classifying the largest number of stages as out of range were: $\bar{NN}$ and $\bar{RMSSD}$ (over 50% of a total number of stages of 20 (1 index × 4 stages × 5 subjects)).

## 4. Discussion

The present study aimed to investigate the ANS responses of healthy individuals during simulated dives in hyperbaric chambers and to explore the effects of humidity and the factors associated with the protocols in both chambers (temperature, body position…) on these responses. Our analysis of HRV-derived indices and their statistical ranges revealed important insights into the ANS responses of the subjects at stages at different depths during the immersions.

The results showed that the $\bar{P_{HF⊥}}$ index was notably reduced in all stages of the humid dataset compared to the dry dataset, indicating a reduction in parasympathetic activity not associated with respiration in humid conditions. This finding is consistent with previous studies [18] and suggests that the longer the time spent within the hyperbaric chamber, the more prominent the sympathetic dominance becomes. Additionally, the $\bar{pNN50}$ index was found to be the most different between datasets, with a reduction in the humid dataset compared to the dry dataset [4]. This points to activation of sympathetic activity triggered by factors such as cold water temperature and psychological stress or anxiety associated with diving. There are, however, studies pointing to an increase in parasympathetic activity when the skin makes contact with water [19] or when subjects breathed through a mouthpiece compared to spontaneous breathing [16]. This effect may also be present in the baseline state, thereby negating its effect when referencing the rest of the measurements to this state. In the case of hyperbaric conditions, there is an additional factor to consider. As the pressure increases, more effort is required to extract air from the regulator, and, over time, this respiratory effort can become tiring, triggering the dominance of the parasympathetic response [16]. All of this may suggest that the impact of the pressure of the environment dominates over other effects. Overall, the impact of the differentiating factors between the dry and humid chambers is mitigated by referencing the indices’ values to those obtained at the baseline stage. For instance, the changes associated with the diving reflex, which typically lead to an increase in parasympathetic activity, as mentioned earlier, already occur during the baseline stage (1D). Consequently, the ANS response stemming from the diving reflex is present throughout all stages. Similarly, the increased vagal tone resulting from breathing through a respiration mask persists throughout the entire immersion period, and referencing the baseline values helps minimize this factor. Furthermore, the decision to keep the divers in a horizontal position in the humid chamber was made to ensure that the entire body experiences the same pressure when immersed in water. If the divers were seated or in a vertical position, there would be variations in the pressure sensed by the head and feet.

One of the key contributions of our study was the classification of subjects into “normal” and “abnormal” categories based on the statistical range between the 5 and 95 percentiles and the IQR for each index and stage. These ranges could be used to generate warnings when monitoring the activity of divers and serve as a reference for avoiding future immersions if many indices are out of the normal ranges. Importantly, all subjects were apparently healthy, and most had significant previous expertise in diving, indicating that these ranges could be used as a baseline for any diver (professional or amateur). Among the few subjects classified as “abnormal” in the dry dataset, the ratio between females and males was much higher than that of the complete dataset (one out of three classified out of IQR). This could indicate that it may be necessary to adapt the warning ranges based on gender differences.

Our analysis also revealed that the humidity of the surrounding environment, together with its associated immersion protocol and body position, plays a crucial role in the ANS response of the subjects and their classification as “abnormal”. The stage with the largest number of indices out of range was “1A”, indicating that the recovery of ANS activity at sea level notably diverges between subjects within the two datasets. The indices with the highest number of stages classified as abnormal with the alternate dataset ranges were different between datasets, with $\bar{NN}$ and $\bar{RMSSD}$ being the most affected in the humid dataset, as well as $\bar{P_{⊥}}$ and, to a lesser extent, $\bar{P_{LF⊥}}$ and $\bar{P_{HF⊥}}$ in the dry dataset. These results suggest that HR, reflected in these time indices, is a crucial factor in determining whether a subject should be warned in a dry immersion, while $\bar{P_{⊥}}$ is key in the classification of abnormality in humid conditions [18].

Finally, we checked whether using mathematical methods to overcome the limitations of the relatively low number of subjects in both hyperbaric chambers by increasing the variability presented by default datasets, such as the bootstrap aggregating method [20], would affect the results of the classification and observed that it had a slight impact on the number of subjects classified as abnormal.

As stated in the Introduction section, the ANS response in variable hyperbaric environments can be influenced by numerous factors, both directly and indirectly. These factors contribute to the complex dynamics of the sympathovagal balance during diving, and gaining a comprehensive understanding of their interplay is crucial. While this study has shed light on some of these factors, there is still a need for further research to delve more deeply into their specific contributions and implications for divers’ physiological responses. One aspect requiring further investigation is the role of some environmental factors, such as water temperature, which may also vary with pressure and humidity. These factors can significantly impact the ANS response and have implications for divers’ cardiovascular and respiratory systems. Examining how changes in these environmental conditions affect the sympathovagal balance will provide valuable insights into the adaptive mechanisms of the ANS during diving.

Additionally, individual factors, including divers’ physical fitness, previous diving experience, and psychological state, can influence the ANS response. Investigating how these individual characteristics interact with environmental factors will enhance our understanding of the personalized nature of the sympathovagal balance in divers. Furthermore, the potential influence of different breathing patterns, respiratory control techniques, and gas mixtures on the ANS response warrants further exploration.

By conducting comprehensive studies that incorporate these various factors, researchers can unravel the intricate web of interactions shaping the sympathovagal balance during diving. This knowledge will not only contribute to our fundamental understanding of human physiology but also have practical implications for diving safety and performance. Ultimately, continued research in this field will provide valuable insights for optimizing dive protocols, training strategies, and medical guidelines tailored to individual divers, enhancing their overall well-being and performance in hyperbaric environments. Overall, our findings provide important insights into ANS responses during simulated dives in hyperbaric chambers and could be used as a reference for divers in the future.

### Limitations

Obtaining an extensive database of signals recorded under realistic diving conditions at such great depths is very challenging for several reasons: specific equipment that is not accessible to the general population is needed (hyperbaric chambers), there are significant health risks for subjects if descent and ascent protocols are not properly followed, and subjects must have prior diving experience. Taking all this into consideration, the number of subjects registered in this study was limited, especially in the dataset from the humid hyperbaric chamber, which could affect the statistical significance of the differences between datasets. Nevertheless, the database was still large enough to derive qualitative conclusions about the impact of humidity on the ANS response during dives.

On the other hand, our study only utilized data from stationary stages with stable barometric pressure lasting several minutes within the immersion. Thus, extrapolating the evolution of index values and their respective ranges during intervals of pressure change should be approached with caution. To further analyze the raw signals and ensure accuracy, non-stationary methods should be considered in future studies.

## 5. Conclusions

The present study explored the ANS responses of healthy subjects during simulated dives in hyperbaric chambers, with a particular focus on the effects of humidity and the associated immersion protocol on these responses. The results demonstrated that subjects in the humid chamber had a significantly different ANS response to immersion, with reduced parasympathetic activity and increased sympathetic dominance in humid conditions. The study also identified several key indices, including the average high-frequency power and the average $\bar{pNN50}$ index, which were particularly sensitive to changes in the humid protocol. These indices were used to classify subjects as “normal” or “abnormal,” with the statistical ranges providing a useful reference for monitoring the health of divers during high-pressure dives. The study also demonstrated the importance of considering the variability in ANS responses among individuals, as well as the impact of the surrounding environment, when assessing the health risks associated with deep sea diving. Overall, the findings highlight the complex interplay between the ANS and environmental factors and the importance of carefully monitoring and managing the health risks associated with high-pressure diving.

## Figures and Tables

**Table 1:** Number and percentage of subjects classified as out of 5–95% range (more than 8 indices out) and IQR (more than 20 indices out), without and with bagging, for dry and humid datasets.

		DATASET		
		DRY (28 Subjects)	HUMID (5 Subjects)	
**DRY RANGE**	**5–95%**	0 (0%)	2 (40%)	(>8 ind. out)
	**IQR**	3 (11%)	3 (20%)	(>20 ind. out)

## Data Availability

The data presented in this study are available on request from the corresponding author.
